# Predicting the Potential for Natural Recovery of Atlantic Salmon (*Salmo salar* L.) Populations following the Introduction of *Gyrodactylus salaris* Malmberg, 1957 (Monogenea)

**DOI:** 10.1371/journal.pone.0169168

**Published:** 2016-12-29

**Authors:** Scott J. Denholm, Andrew S. Hoyle, Andrew P. Shinn, Giuseppe Paladini, Nick G. H. Taylor, Rachel A. Norman

**Affiliations:** 1 Integrative Animal Sciences, Animal & Veterinary Sciences Research Group, Scotland’s Rural College (SRUC), Edinburgh, United Kingdom; 2 Computing Science and Mathematics, School of Natural Sciences, University of Stirling, Stirling, United Kingdom; 3 Institute of Aquaculture, School of Natural Sciences, University of Stirling, Stirling, United Kingdom; 4 Centre for Environment, Fisheries & Aquaculture (CEFAS), Weymouth Laboratory, Weymouth, United Kingdom; Swansea University, UNITED KINGDOM

## Abstract

*Gyrodactylus salaris* (Monogenea, Platyhelminthes) is a notifiable freshwater pathogen responsible for causing catastrophic damage to wild Atlantic salmon stocks, most notably in Norway. In some strains of Baltic salmon (*e*.*g*., from the river Neva) however, the impact is greatly reduced due to some form of innate resistance that regulates parasite numbers, resulting in fewer host mortalities. *Gyrodactylus salaris* is known from 17 European states; its status in a further 35 states remains unknown; the UK, the Republic of Ireland and certain watersheds in Finland are free of the parasite. Thus, the parasite poses a serious threat if it emerges in Atlantic salmon rearing regions throughout Europe. At present, infections are generally controlled via extreme measures such as the treatment of entire river catchments with the biocide rotenone, in order to remove all hosts, before restocking with the original genetic stock. The use of rotenone in this way in EU countries is unlikely as it would be in contravention of the Water Framework Directive. Not only are such treatments economically and environmentally costly, they also eradicate the potential for any host/parasite evolutionary process to occur. Based on previous studies, UK salmon stocks have been shown to be highly susceptible to infection, analogous to Norwegian stocks. The present study investigates the impact of a *G*. *salaris* outbreak within a naïve salmon population in order to determine long-term consequences of infection and the likelihood of coexistence. Simulation of the salmon/ *G*. *salaris* system was carried out via a deterministic mathematical modelling approach to examine the dynamics of host-pathogen interactions. Results indicated that in order for highly susceptible Atlantic strains to evolve a resistance, both a moderate-strong deceleratingly costly trade-off on birth rate and a lower overall cost of the immune response are required. The present study provides insights into the potential long term impact of *G*. *salaris* if introduced into *G*. *salaris*-free territories and suggests that in the absence of external controls salmon populations are likely to recover to high densities nearing 90% of that observed pre-infection.

## Introduction

*Gyrodactylus salaris* Malmberg, 1957 is a viviparous (*i*.*e*., live-bearing) freshwater ecto-parasite that infects both wild and farmed populations of Atlantic salmon (*Salmo salar* L.), potentially resulting in juvenile host mortality. It is an Office International des Epizooties (OIE) listed pathogen that was first described from the fins and skin of a Baltic Atlantic salmon strain from a hatchery in Sweden located near the Indalsälv river [1]. The parasite is believed to be native to the waters of northern Russia, western Sweden and northern Finland [2], but is now known to be widely distributed throughout Europe [3–10] and recently confirmed in Romania [11]. In Norway, the parasite has caused catastrophic damage to wild populations of Atlantic salmon parr since it was first observed the mid-1970s after a period of mass salmon mortality [12–15]. Moreover, this parasite is known to have been introduced to Norway on at least three separate occasions [16] and can reduce salmon stock in rivers by approximately 85% on average [10]. Within 5 years of initial introduction to a susceptible host population reductions in outbound smolts can be as high as 98% [10,12,17]. This has caused severe damage to the Norwegian economy and to wild salmon fisheries. Although infections in salmon hatcheries have been reported, such infections are more readily controlled, however, if left untreated salmon mortality can reach 100% [10]. In the years post introduction, *G*. *salaris* has been reported from 50 rivers, 13 Atlantic salmon hatcheries and 26 rainbow trout (*Oncorhynchus mykiss* Walbaum) hatcheries in Norway and subsequently managed through coordinated intervention [18]. Subsequent losses to the Norwegian salmon industry up until 2004 exceeded US$ 655m [19]. The last time loss figures were estimated annual loss of wild juvenile salmon was suggested to be in the region of 250–500 metric tonnes as a consequence of parasitic infection reducing the average density of salmon parr in infected rivers [19]. Such annual loss costs the Norwegian economy over US$ 55m per annum through surveillance and eradication (circa US$ 23m per annum) along with losses incurred by fisheries, associated industries and tourism (circa US$ 34m per annum) [14]. Hence, *G*. *salaris* poses a serious threat if it establishes in territories that are currently *G*. *salaris* free [9].

Though *G*. *salaris* has had a huge impact in Norway, some Baltic strains of Atlantic salmon appear to be more resistant to the parasite than the Atlantic strains [19]. Bakke *et al*. [20] was the first study to show a difference in the immune response between two strains of salmon. In particular, they showed that parasite numbers grew exponentially on individual fish from an Atlantic strain of Atlantic salmon from the rivers Lone and Alta (Norway), whereas on a Baltic strain of Atlantic salmon from the river Neva (Russia) there was some initial growth in parasite numbers, but those numbers peaked and then generally decreased to zero. This clearly demonstrated some differences in susceptibility of these salmon strains to *G*. *salaris* through the ability of the some Baltic strains to exhibit some form of resistance or immune response [19–23]. It has been highlighted that the resistance observed in some Baltic salmon strains, such as those from the Neva river, is due to the presence of the parasite in the Baltic watershed since the last glacial period allowing an evolutionary selection process within the host [22]. This supports the hypothesis that *G*. *salaris* is a recent (c. 40 years) introduction to Norwegian rivers and potentially explains why Norwegian Atlantic salmon are particularly susceptible to the parasite.

Due to the impact of *G*. *salaris* on Norwegian salmon, extreme measures have been taken to try and control and eradicate the parasite. These measures include the treatment of entire river catchments with the biocide rotenone [24] to remove all hosts (and hence, *G*. *salaris*), before restocking with the original genetic stock [12,14,25,26]. The use of rotenone in this way in EU countries is unlikely as it would be in breach of the Water Framework Directive [27]. Not only are such treatments economically and environmentally costly, they also eradicate the potential for any host/parasite evolutionary process to occur.

Currently the only European countries recognised as free from *G*. *salaris* infection are the United Kingdom [28,29], the Republic of Ireland [9,30,31], and some areas of Finland [9,32]. Other countries such as Portugal, Spain and France, where *G*. *salaris* has been previously recorded, are believed to be misidentifications with a morphologically similar species *Gyrodactylus teuchis* Lautraite, Blanc, Thiery, Daniel et Vigneulle, 1999 [32,33]. The collection of further material from these states is required to determine their current *G*. *salaris* status. Recently, however, it was proposed that *G*. *salaris* and *G*. *thymalli* Žitňan, 1960, another morphologically similar and closely-related, but benign parasite of grayling, *Thymallus thymallus* L., may represent a single species of *Gyrodactylus* that comprises several pathogenic and non-pathogenic strains on a number of primary hosts [34]. The study [34] analysed microRNA loci from a small number of populations of *Gyrodactylus* from Atlantic salmon and grayling hosts and made the proposal that the two species should be synonymised, however, this has not yet been formally accepted by the OIE and as such this synonymisation is yet to be accepted by the scientific community [11].

Despite the fact *G*. *salaris* is not present in the UK but *G*. *thymalli* is, it has been demonstrated that UK salmon populations have similar levels of susceptibility to infection as those in Norway [15,23,35,36]. Due to this, *G*. *salaris* is regarded to pose a serious disease threat to the UK’s valuable wild and farmed salmon populations [37]; a report to the Scottish Government advised if *G*. *salaris* were introduced into Scotland, as an example of potential impact, then the potential losses would be estimated at £44.8 million per annum to the Scottish economy, £34.5 million to Scottish household income each year and 1,996 full time equivalent jobs lost in Scottish employment [38]. It is also likely that *G*. *salaris*, if introduced, would spread within and between UK rivers before it is detected [2]. Due to this, contingency plans were drawn up setting out a series of actions to follow in the event of an outbreak [37]. Using mathematical modelling approaches based on the existing knowledge of *G*. *salaris*, the present study aims to simulate salmon/*G*. *salaris* interaction dynamics in order to investigate the potential for natural recovery of susceptible salmon populations post introduction of *G*. *salaris* infection.

The majority of previous mathematical modelling work concerning the salmon/*G*. *salaris* system has been centred on risk and statistical analysis highlighting areas such as routes of infection, transmission and risk of introduction [2,39–43]. Some work has been carried out to study the effects of *G*. *salaris* on different stages of the salmon life-cycle [44] as well as the effect of other gyrodactylid species such as *Gyrodactylus turnbulli* Harris, 1986 on guppies, *Poecilia reticulata* Peters, 1859 [45,46]. More recently stochastic models have become popular in studying *G*. *salaris* infections in salmon and modelling techniques such as Leslie matrix population models and individual based models have also been employed [47–49]. Though a great deal of effort has been placed on understanding the risks and routes by which the parasite may be introduced, little has been done to predict its long-term impact. Moreover, not much is known about what may happen should control efforts similar to those employed in Norway not be possible.

In the present study a series of host-macroparasite models are developed, first considering a single fish host and incorporating that into a population model. The effects that an increased immune response has on the host and parasite populations are analysed demonstrating the difference in susceptibility between a highly susceptible salmon strain and a resistant strain. Finally, some mutation and replacement is incorporated to determine how strong an immune response the hosts develop and what types of trade-offs and parameter values are required to allow a fully susceptible host to evolve into a primarily resistant host.

## Methods

### Individual fish model

To model parasite numbers on an individual host a deterministic ordinary differential equation (ODE) approach is taken. For the number of parasites, *P*, a simple exponential growth model is assumed, with replication rate *μ*, death rate *ε* and dislodgement rate *λ*. In addition, we include an immune response, *I*, exhibited by the host which activates at rate *m* as parasite numbers grow; this in turn increases the parasite death rate by a rate *ρI*. Finally, the immune response decays at a continuous rate *ξ*. The equations for these are shown in Eq (1) below:

$$\begin{array}{lll} \frac{dP}{dt} & = & P\left(\mu -\varepsilon -\rho I-\lambda \right)\\ \frac{dI}{dt} & = & mP-\xi I \end{array}$$ (1)

### Full salmon population model

The individual fish host model was expanded by scaling up the equations in Eq (1), to a population of hosts and parasites. Here the host population, *H*, is assumed to follow a logistic growth function, *a* being the birth rate, *b* the natural death rate and *s* representing density-dependent competition, with an additional death rate dependent on parasite burden, *αM*. The equations for average parasite burden, *M = P/H*, or density of parasite per host (where *P* is the total on-host parasite density), and immune response, *I*, are taken from Eq (1), but expanded in that the parasite burden decreases due to deaths of the host due to infection, *α*, and birth of new (initially parasite-free) hosts. The on-host parasite distribution is assumed to follow a Poisson distribution across the host population, which is taken into account in the parasite-induced death rate, *α*. Both Poisson and negative binomial distributions were considered with each giving similar results, the Poisson however, simplified the model significantly and thus was chosen. The off-host parasite density, *W*, is assumed to increase as the parasites leave the host (either by choice or host death) and decrease due to parasite death, *σ*, or parasite latching on to hosts at a rate *β*, which in turn increases parasite burden. It is important to note that actual parasite death rates are highly dependent on many factors such as environmental conditions (*e*.*g*. temperature), water quality, salinity, *etc*. [50,51]. In the present study, however, we consider a simplified worst case scenario such that we have a highly pathogenic strain of parasite and a highly susceptible Atlantic salmon strain.

The dynamics for the model take the form in Eq (2). Further details of the model’s derivation are presented in the Supplementary Information (S1 Appendix). Parameter values used in all models are given in Table 1. Parameter values regarding the UK were used where available.

$$\begin{array}{lll} \frac{dH}{dt} & = & \left(a-b-sH\right)H-\alpha MH\\ \frac{dM}{dt} & = & \left(\mu -\varepsilon -\rho I-\lambda -\alpha -a\right)M+\beta W\\ \frac{dI}{dt} & = & mM-\xi I\\ \frac{dW}{dt} & = & MH\left[\lambda +b+sH+\alpha \left(1+M\right)\right]-\sigma W-\beta WH \end{array}$$ (2)

**Table 1:** List of parameter values used to inform salmon/*G*. *salaris* host parasite models.

Parameter	Description	Estimate/day	Source
***a***	Maximum salmon birth rate	0.02	Assumed
***b***	Salmon natural death rate	0.00057	[52]
***K***	Salmon carrying capacity	0.125	[52]
***s***	Density dependent constraint	0.000155	Estimated using *K* for 1000 m^2^
***μ***	*G*. *salaris* birth rate (Norway)	0.1825	[20]
	*G*. *salaris* birth rate (UK)*	0.1708	[15]
***ε***	*G*. *salaris* on-host death rate	0.08	[50]
***σ***	*G*. *salaris* off-host death rate	0.14–0.17	[42]
***λ***	Rate the parasites leave the hosts	0.06	Assumed
***β***	Parasites attach rate to hosts	0.0585	Assumed
***α***	Parasite induced death rate of host	0.02	[45]
***m***	Rate hosts develop an immune response	0–0.0175	Assumed
***ξ***	Decay rate of immune response	0.0055	Assumed
***ρ***	Rate of increase in parasite mortality due to resistance	1	Adjusted in values of *m*

* parameter value used in this study

With macro-parasite models, such as those used in the present study, fish-to-fish transmission is not shown explicitly in the model, but is rather an implicit feature modelled through the distribution of parasites across the fish population. This is due to the fact that *P* gives the total number of on-host parasites which remains unchanged as parasites switch between fish hosts, and due to the large number of parasites involved in these systems, the effect on the distribution of parasites is negligible.

## Results

### Single host model

**Fig 1:**
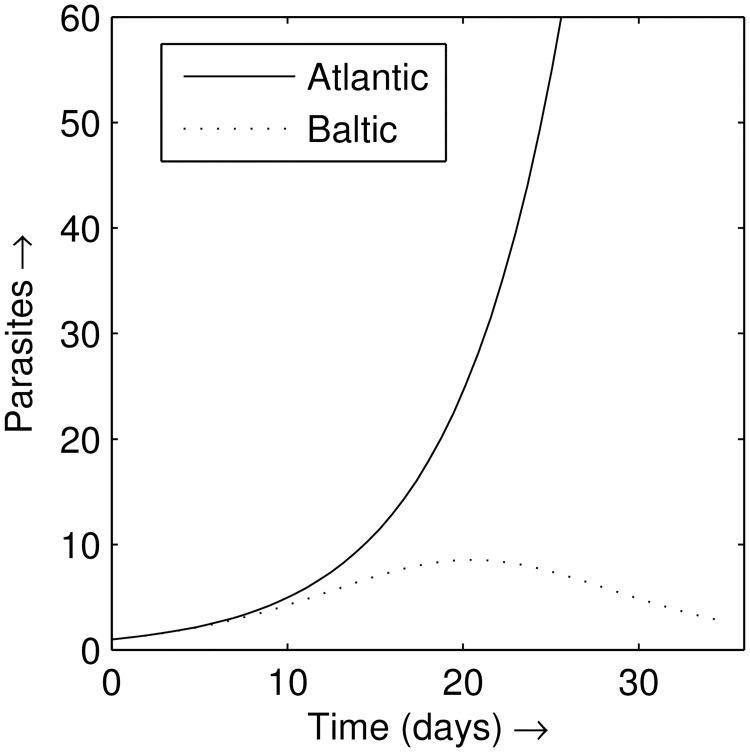
Output from the model in Eq (1) for parasite numbers, with *m* = 0 (susceptible salmon strain—solid line) and *m* > 0 (resistant salmon strain—dashed line).

Using the single host model, Eq (1), two different cases were considered (Fig 1): firstly, a highly susceptible Atlantic salmon strain with no immunity, *m* ≈ 0; secondly, a resistant salmon strain, *m >* 0 (*m* = 0.0175). Model simulations showed parasite numbers grew exponentially on the susceptible host, whereas on the resistant host parasite numbers decayed to zero. In the case of the resistant host initial parasite growth over the first 7 days was similar to the highly susceptible host, however, parasite population growth slowed thereafter, peaking at around 20 days, before decreasing to zero/low levels. These behaviours approximately follow the experimental results observed by Bakke *et al*. [20] at water temperatures of 12°C on Atlantic Lone and Baltic Neva salmon hosts.

### Full salmon population model

**Fig 2:**
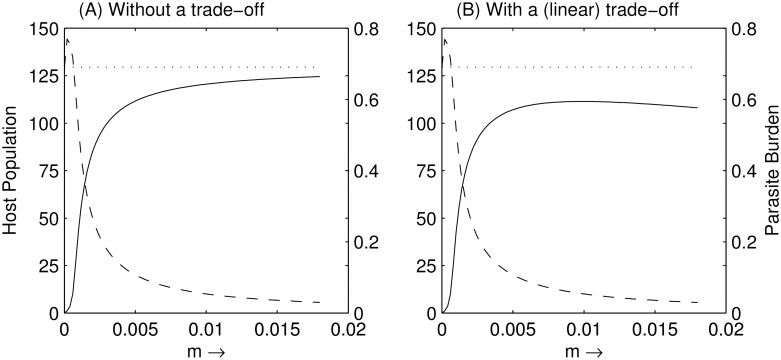
Plot of host (equilibrium) population *H* (solid line) and parasite burden *M* (dashed line). (A) with no trade-off; (B) with a linear trade-off on host birth rate. The dotted line represents the (fully susceptible) host population before the parasite outbreak.

Firstly, the model in Eq (2) was simulated to consider a fully susceptible host with a negligible immune response, *i*.*e*. *m* ≈ 0. Here, following the introduction of the parasite into the system the model shows a fast drop in the number of hosts. This mirrors the results in the field, *e*.*g*., in Norway where the parasite can reduce the salmon parr population by up to 98% within 5 years [12]. As host extinction has not been witnessed, and the average reduction in salmon is 86% (and sometimes lower), we can assume that although *m* ≠ 0, it must be very small. As we increase the amount of immune response, *m* (Fig 2A), we very quickly see that the host (equilibrium) population recovers and the average parasite burden decreases. In fact, only negligible values for *m* produces a reduction approaching 100%, and even a small amount of resistance significantly improves host population size. Moreover, host numbers approach their pre-infection levels, and parasite burden approaches zero, as *m* gets large. Interestingly the greatest effect on host and parasite numbers occurs at lower increases in immune response *m*, with only marginal effects for larger *m*.

### The trade-off

So far we have assumed that the immune response mounted by the host is cost free. This, however, has been shown not to be the case. One prime example of this is a study of furunculosis in brook trout, *Salvelinus fontinalis* (Mitchill) [53], in which it was shown that an increase in immunity had a negative effect on the host’s birth rate; they observed approximately a 7 to 12% decrease in the birth rate of the trout that exhibited resistance to infection. Although there is no evidence to support or deny that a similar trade-off exists in salmon, for the remainder of this study we hypothesise there is a cost of the immune response. In particular, we take a trade-off such that the development of an effective immune response, as measured here by *m*, can have a significant negative effect on host birth rate *a*, such that *a = a(m)* with *a’(m) < 0*. Although the form of *a(m)* is unknown, we make two assumptions: i) when *m* = 0, *a* = 0.02 (maximum birth rate) representing a highly susceptible salmon strain, and ii) when *m* = 0.0175 (our maximum resistance), birth rate *a* is reduced by 10% representing a resistant salmon strain. We initially take a linear trade-off (straight line) passing through these two points to allow us to interpolate *a* for intermediate *m*.

The addition of this trade-off has a marked effect on the host population. In particular, at high levels of immune response, *m*, the cost of a lower birth rate begins to outweigh the benefit of higher immune response (and subsequent lower parasite burden) and the host population begins to decrease (Fig 2B). Here an optimal level of immunity now exists which maximises the host population when *m* = 0.010.

### Mutation and replacement of hosts

The optimal immune response observed may not, however, represent the level of *m* that the host species evolve to; this instead would likely be determined by the level of *m* which optimises the growth rate of the host population. To study the long-term evolution of immune response, we take a mutation and replacement approach, broadly following that of adaptive dynamics [54].

Consider a single resident host strain of salmon, with immune response *m* and population density *H* existing alone in an environment, with the dynamics as given in Eq (2). Now suppose a mutation creates a host with slightly different immune response $\hat{m}$, with population density $\hat{H}$. Mutations are generally small, and hence, the difference between *m* and $\hat{m}$ is small. Here $\hat{M}$ and $\hat{I}$ are the (average) parasite burden and immune response for this mutant host strain. If this new type is initially rare, then we can write down the fitness of this mutant type, *i*.*e*. the long-term growth rate of this mutant population, as

$$r\left(\hat{m},m\right)=a\left(\hat{m}\right)-b-sH\left(m\right)-\alpha \hat{M}\left(\hat{m},W\left(m\right)\right)$$ (3)

Here $\hat{M}$ is the average parasite burden on a mutant host. We make the assumption that parasites will reach their “average” (equilibrium) burden on the new mutant host type $\hat{M}$ quickly, when compared to the natural fish lifespan—a reasonable assumption given the much shorter generation time of the parasite. The full derivation of the fitness is given in the Supplementary Information (S3 Appendix). If the fitness is positive, then the mutant host type will increase in number, generally replacing the existing resident host type, whereas if the fitness is negative the mutant will die out. For simplicity, we assume no ‘intermediate strains’ due to cross-breeding. The fitness is used to calculate the location of the evolutionary singular point and determine whether it is an evolutionary steady state, ESS, *i*.*e*. an evolutionary end point.

**Fig 3:**
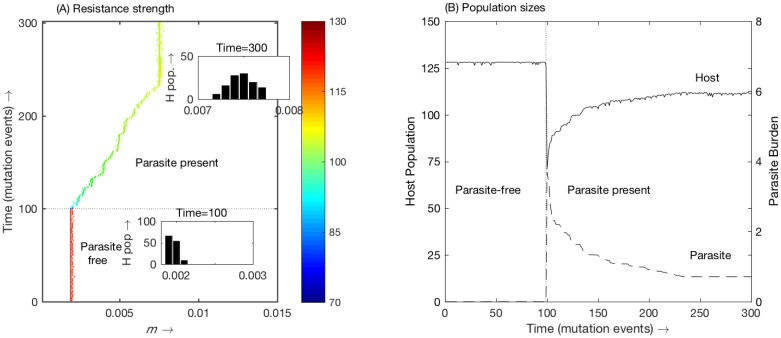
In (A) we plot how *m* evolves over time, with a linear trade-off; the colour of the line denotes the total host population at that time. The inset graphs give the distribution of resistance levels in the host population at time = 100, just prior to parasite invasion, and at time = 300, when the population reaches its ESS. In (B) we plot the host population and parasite burden over time, corresponding to *m* evolving.

To demonstrate the evolutionary behaviour more clearly, we numerically simulate evolution using a similar mutation and replacement approach, using the full mutant-resident dynamics—details of which are presented in the Supplementary Information (S3 Appendix). This has been shown to be a good approximation to the analytical approach using the fitness in Eq (3) and has the benefit of not making the assumption about the parasite burden being at equilibrium. Starting from a highly susceptible salmon strain, we plot how *m* evolves through time. Fig 3A plots the strains present following each mutation and shows how *m* evolves over time with a (linear) trade-off. Here ‘time’ means the number of mutation events that occur—as we do not currently know how often mutations occur, we leave time deliberately in terms of these mutation events. In addition, the colouring represents the total host population present. For the first 100 time steps, the system is parasite-free, hence minimal resistance and maximum host population (Fig 3A). At time step 100, however, we introduce a small number of (free-living) parasites. Immediately the population of host drops (Fig 3B). Resistance then begins to be selected for, leading to an increase in *m* (Fig 3A). This in turn leads to an increase in host population and a lower parasite burden (Fig 3B). The level of immune response eventually settles at an intermediate level, *i*.*e*. an ESS, with the host population normally distributed about this resistance level (Fig 3A- inset). This is at approx. *m* = 0.0075 here, slightly below the optimal *m* (≈ 0.010) which maximises the host population.

### Trade-off shape

So far we have only considered a linear trade-off—whereby each benefit (*i*.*e*. unit increase in immune response, *m*) always comes at the same cost (*i*.*e*. same decrease in birth rate, *a*). We now vary the trade-off shape by means of a parameter *θ* (see Supplementary Information, S3 Appendix, for specific details). Specifically, a positive *θ* represents an ‘acceleratingly costly trade-off’, whereby each benefit comes at an increasing (accelerating) cost (*i*.*e*. larger decrease in birth rate, *a*); with larger *θ* giving a greater effect. Conversely, a negative *θ* represents a ‘deceleratingly costly trade-off’, whereby each benefit comes at a decreasing (decelerating) cost (*i*.*e*. a smaller decrease in birth rate, *a*). Finally *θ* = 0 represents a linear trade-off [54].

**Fig 4:**
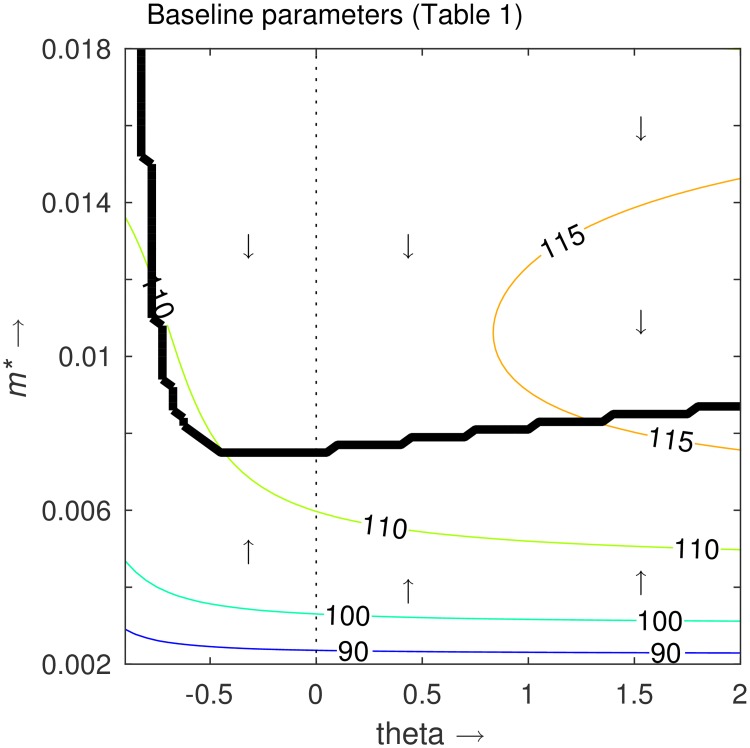
Plot of the evolutionary singular point (ESS—thick black line) for various shapes of trade-off. Here *θ <* 0 represents a deceleratingly costly trade-off; *θ >* 0 represents an acceleratingly costly trade-off; and *θ* = 0 (dashed line) represents a linear trade-off—as taken in Fig 3 simulation. The host evolves such that the immune response *m* either increases or decreases (vertically on the plot) to the singular point—see Supplementary Information (S4 Appendix) for derivation of this line. The thin contour lines represent the total host population size for corresponding values of *m* and *θ*. The parameters are as given in Table 1.

In Fig 4 we plot how the evolutionary singular point (ESS) m* changes as we change the shape of the trade-off (*θ* values); where the ESS is denoted by the thick black line. The host evolves to increase their resistance level *m* if currently below the ESS, and evolve to decrease resistance if above. In addition, the contour lines represent the equilibrium host density. We immediately gain two main results from this. Firstly, that the evolutionary singular points are always just below the maximum host density for each specific value of *θ*, meaning that the optimal value of *m* which maximises the host population is not the same value of *m* that maximises host fitness. Secondly, for strong deceleratingly costly trade-offs, as θ→ -1, the host evolves to maximise the immune response *m*, whereas for weakly deceleratingly costly or acceleratingly costly trade-offs, the host evolves to an intermediate value of *m*. This suggests a limited range of trade-offs that allow a highly susceptible salmon host strain to evolve into a highly resistant host strain.

#### Virulence

**Fig 5:**
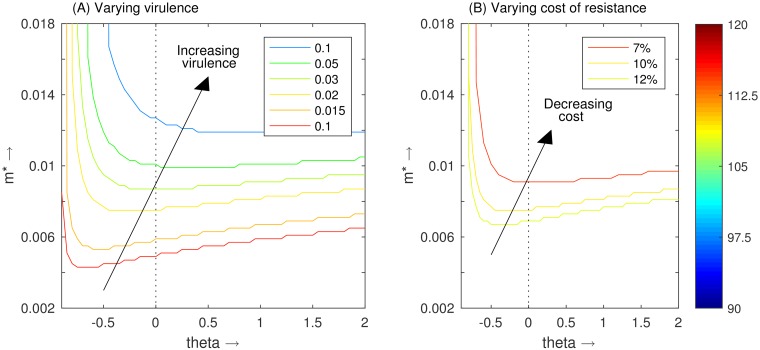
Plot of the evolutionary singular point (ESS) for various shapes of trade-off: *θ <* 0 deceleratingly costly, *θ >* 0 acceleratingly costly and *θ* = 0 (dashed line) linear. The colour of each line is defined by the average host density along that line, as represented on the colour bar. In (A) the virulence of the pathogen is varied, with *α* = 0.02 being the baseline value. In (B) the cost of resistance is varied, with 10% being the baseline.

In Fig 5A, we plot the evolutionary singular points (ESS) for varying levels of parasite virulence, in terms of a higher or lower parasite-induced host death rate, *α*. Higher levels of virulence, common in *G salaris* [15,23,35,36], encourages the evolution of a stronger immune response.

#### Cost of resistance

In Fig 5B, we show the equivalent results for the lower and upper estimates for the cost of resistance, as given by Cipriano *et al*. [53], 7% and 12% respectively (as opposed to the ‘averaged’ 10% initially taken). As would be expected, the location of the evolutionary singular points (ESS) is lowered as the cost of resistance is increased, implying that the hosts evolve a lower immune response, *m*, if more costly. This suggests that for the host to evolve into a highly resistant strain, the cost of being highly resistant must not be too high.

## Discussion

Wild Atlantic salmon populations the world over are currently threatened, with numbers in some regions in decline [55]. The catastrophic impact that infections by *G*. *salaris* can have on susceptible salmon populations, and the consequential financial implications, have already been witnessed in Norway [12,17,56,57]. In the years post introduction to Norway, *G*. *salaris* has since been reported from many other river systems throughout Europe [3–8,10]. The aim of the present study was to explore the long-term interactions between populations of Atlantic salmon and the monogenean parasite *G*. *salaris* in order to make predictions on the natural recovery of salmon populations post introducing such an infection into an environment containing susceptible salmon host populations such as the United Kingdom.

In the present study models were used to study the possible differences between strains of Atlantic salmon to determine the mechanisms evolved by some Baltic strains in order to be able to beat infection and in some cases coexist with low levels of *G*. *salaris* infection. Model outcomes have highlighted that simple host-parasite models can show the varying levels of resistance as seen in the Atlantic Lone and Baltic Neva salmon systems, with the addition of an immune response. Models were used to investigate the possibility highly susceptible strains of Atlantic salmon evolving traits and resulting trade-offs to become more like their resistant counterparts.

Results from the present study highlight salmon will evolve to a more resistant state and therefore be able to naturally recover from *G*. *salaris* infection if the salmon immune response is allowed to evolve. This evolution would be subject to a trade-off such that host birth rate is negatively correlated with resistance. Such recovery would result in host coexistence, potentially at relatively high host densities, nearing 90% to that observed in the absence of infection, with low parasite densities. The level of immune response however depends on several factors: In order for a susceptible host to gain the level of resistance witnessed in some Baltic salmon strains, it requires both a moderate-strong deceleratingly costly trade-off (*i*.*e*., the host pays a large cost in the creation of the immune response, for low *m*, and then the additional costs for improving that immune response, increasing *m*, are less and reducing) and a lower overall cost of the immune response. In addition, the virulence of parasite can play a significant part, with higher virulence rates leading to lower host population sizes but higher resistance levels; conversely, lower virulence rates leads to higher host populations with lower resistance levels. For this reason, the water chemistry can play a crucial part in how salmon evolve as identical strains of parasite can have different virulence rates solely due to environmental factors.

In general, mathematical models represent a simplified version of a system, as such, there are always going to be certain limitation. Future studies would do well to build on the models herein and explicitly model the seasonal effects and implications of the salmon and gyrodactylid life-cycles. Salmon spawning, for example, primarily takes place once a year between mid-October and late February [58]. Similarly, salmon do not spend their entire life in a river and in fact spend the majority of their adult life at sea, returning to their natal river to spawn. Though it is possible for some salmon parr to mature sexually in a river without the need to run to sea, and hence, stay to participate in spawning [59]. Such behaviours will have an important impact on the length of time it would take for a population of salmon to recover from *G*. *salaris* infections due to the time between salmon leaving and returning to infected rivers.

Salinity and water temperature are very important in determining *G*. *salaris* survival. *Gyrodactylus salaris* is a freshwater parasite and survival is only possible in waters with a salinity between 0–20ppt at temperatures of 3°C—20°C [50,51]. The survival of *G*. *salaris* in low salinity waters has been shown to be negatively correlated with water temperature and hence, parasites can survive longer, both on and off a host, in such waters at lower temperatures [51]. Environment can also play an important role; in situations where water velocity is high, detached parasites have the potential to drift further down a river and infect new populations of hosts. Infection may also have an impact on the way in which salmon interact with each other, for example, in populations of guppies, *P*. *reticulata*, (where individuals are infected with *Gyrodactylus turnbulli*) females have been observed preferring, and selecting, males with low parasite burdens [60]. Furthermore, changes in host feeding behaviour has also been witnessed with feeding response and feeding activity significantly negatively correlated with parasite load [61].

Whilst the varying degrees of pathogenicity of the different *G*. *salaris* strains was not explicitly modelled in the present study, future studies would do well to include such information into predictive models. Different strains of *G*. *salaris* have been shown to have varying effects on salmon hosts [16]. The three currently known clades of *G*. *salaris* include *G*. *salaris sensu stricto*—a highly pathogenic strain only found on Atlantic salmon (Clade I); a strain found on salmon from the river Göta älv in Sweden (Clade 2); and a strain that was found on salmon from the rivers Lærdalselva, Drammenselva and Lierelva in Norway and on rainbow trout from a fish farm in Lake Bullaren, Sweden [16]. A further strain of *G*. *salaris* has been found on rainbow trout in Denmark [3,4]. This variant of the *G*. *salaris* parasite shows low virulence towards Atlantic salmon and under experimental conditions, on isolated hosts, this strain showed limited reproduction or no establishment at all [62]. Lindenstrom et al. [63], however, observed high susceptibility to this strain in rainbow trout and noted that this strain of the parasite greatly resembles *G*. *salaris sensu stricto*.

As highlighted earlier, fish-to-fish transmission was modelled through the distribution of parasites across the fish population and not as an explicit feature in the model. The models proposed consider the total densities of a *G*. *salaris* population within a salmon host population. It would also be interesting to take an approach looking into the density of *G*. *salaris* populations on individual hosts within a population with particular focus on the impact that fish-to-fish transmission has on the dynamics of infection. It is known that juvenile Atlantic salmon are highly territorial [59,63] and hence have a high chance of becoming infected due to fish-to-fish contact when defending a territory against an infected individual. Moreover, fish-to-fish contact between dead infected hosts and live uninfected hosts as well as live infected hosts and live uninfected hosts also provide important routes for *G*. *salaris* spread [64,65].

Aggregation of parasites on hosts also has an important impact on the evolutionary and population dynamics of both parasites and hosts [66,67]. Many studies have been carried out in this area in order to develop our understanding of what causes heterogeneity in the distribution of macroparasites within a host population [68]. Parasite aggregation in the wild is often complex, in macro-parasitic infections the majority of hosts are observed harbouring a low number of parasites with a minority of hosts harbouring a large number [69]. Such skewed aggregations have been shown to follow a negative binomial distribution [66,67,69]. The negative binomial distribution, (defined as *s*^*2*^ = *m* + *m*^*2*^*/k*, where *s*^*2*^ and *m* are the variance and mean respectively) quantifies the (inverse) degree of aggregation via the parameter *k* [70] such that for small *k* parasite aggregation is increased, whereas for large *k* aggregation decreases. The negative binomial distribution converges on the logarithmic series as *k→0* and on the Poisson for *k ≳ 20* [68,71]. Due to the complicated life-cycle of *G*. *salaris* and its similarities with micro- as well as macro-parasites we used a Poisson (defined as *s*^*2*^ = *m*) to model parasite aggregation in the present study, thus, allowing parasites to be randomly (and evenly) distributed throughout the host population. This simplified model analyses considerably whilst still allowing for important observations to made on the dynamics of infection. Previous studies have considered a Poisson distribution when modelling free-living *G*. *salaris* parasites [72]. Moreover, the effect on the distribution of parasites is negligible due to the large number of parasites considered in the present models.

Even though the literature concerning *G*. *salaris* infections in salmon is vast, models would greatly benefit from more accurate and up to date parameter estimates. Experimental studies undertaken exclusively for this reason would be worthwhile in order to obtain estimates for currently unknown parameters. Through our research we have determined that more data are required in order to accurately parameterise the rate at which parasites leave, attach to and kill hosts.

At present the United Kingdom and Ireland are the only known countries to officially establish complete freedom from *G*. *salaris* infections [10,28–30,37]. As highlighted earlier, Atlantic salmon populations in the UK are believed to be just as susceptible as those found in Norway [15,35], hence, if *G*. *salaris* was introduced a similar environmental impact to that of Norway can be expected. Extreme measures have been adopted in an attempt to control and eradicate *G*. *salaris* infections. While eradication is preferred, this rarely happens and hence “management and control” is what is actually being carried out and alternative methods of treatment such as aluminium have been trialled [73]. It is understandable that survivors are undesirable as we may see the development of resistance in the parasite population with consequentially continued catastrophic effects on the host population, however, we also would like to see the evolutionary process occur where there is adaptation or co-evolution to the extent that parasite and host to co-exist without mortality and parasite numbers are maintained at low levels or are removed by the host. Our results highlight that the current practice of treating entire river catchments with rotenone before restocking with salmon from the original genetic stock [12,14,25,26] may be severely damaging the potential for any evolutionary process to occur.

Results from the present study have provided evidence that in the absence of intervention salmon populations should naturally recover from *G*. *salaris* infection, however, the timescale required for this to happen remains unknown. Furthermore, model output suggests susceptible populations would evolve such that they reach a level of resistance required to coexist with the parasite and recover to relatively high densities, nearing 90% of that observed pre-infection. *Gyrodactylus salaris* and its impact on susceptible hosts must continue to be studied in order to aid in contingency planning and defence against introduction and emergence.

## Supporting Information

S1 Fig Schematic representation of salmon-*Gs* model. (TIF)

S2 Fig The trade-off between host birth rate, *a*, and the rate hosts mount an immune response to the parasite (resistance), *m*. (TIF)

S1 Appendix Derivation of the model. (DOCX)

S2 Appendix Equilibrium and stability analysis of model. (DOCX)

S3 Appendix Derivation of the fitness of mutant type and trade-off. (DOCX)

S4 Appendix Details of evolutionary simulations. (DOCX)

S5 Appendix Supporting Information reference list. (DOCX)
