# Correction: Predicting the Potential for Natural Recovery of Atlantic Salmon (*Salmo salar* L.) Populations following the Introduction of *Gyrodactylus salaris* Malmberg, 1957 (Monogenea)

**DOI:** 10.1371/journal.pone.0172497

**Published:** 2017-02-14

**Authors:** Scott J. Denholm, Andrew S. Hoyle, Andrew P. Shinn, Giuseppe Paladini, Nick G. H. Taylor, Rachel A. Norman

The following information is missing from the Funding section: SJD is supported by the Biotechnology and Biological Sciences Research Council (BBSRC grant number BB/K002260/1).
