# Supplementary figures and images for: Predicting the Potential for Natural Recovery of Atlantic Salmon (Salmo salar L.) Populations following the Introduction of Gyrodactylus salaris Malmberg, 1957 (Monogenea)
Source: PLoS One. 2016 Dec 29;11(12):e0169168. doi: 10.1371/journal.pone.0169168 (PMC5199095; doi:10.1371/journal.pone.0169168)

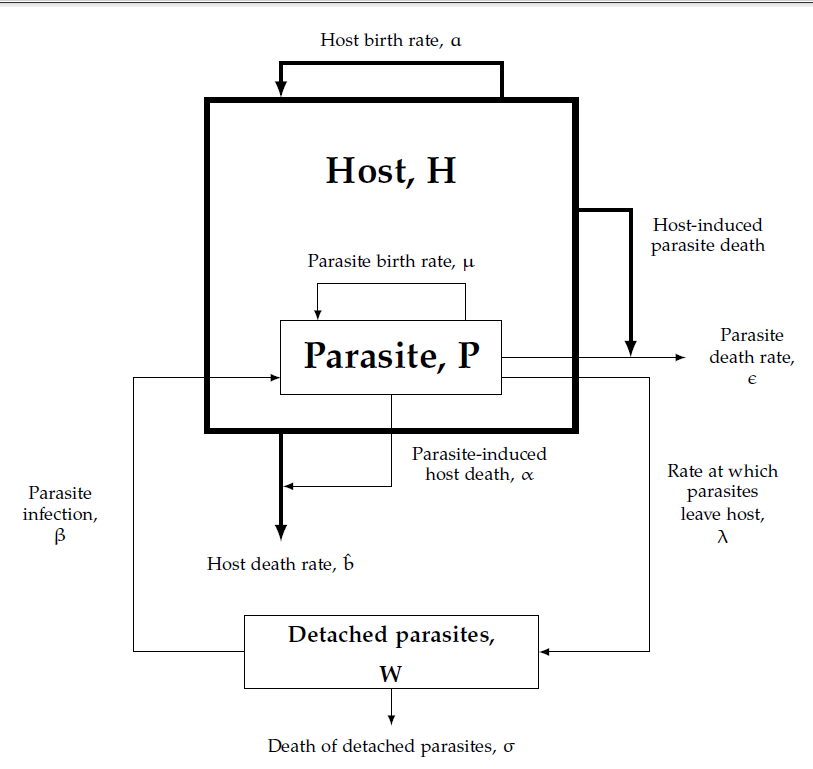

Supplement: S1 Fig — (TIF) [file pone.0169168.s001.tif]

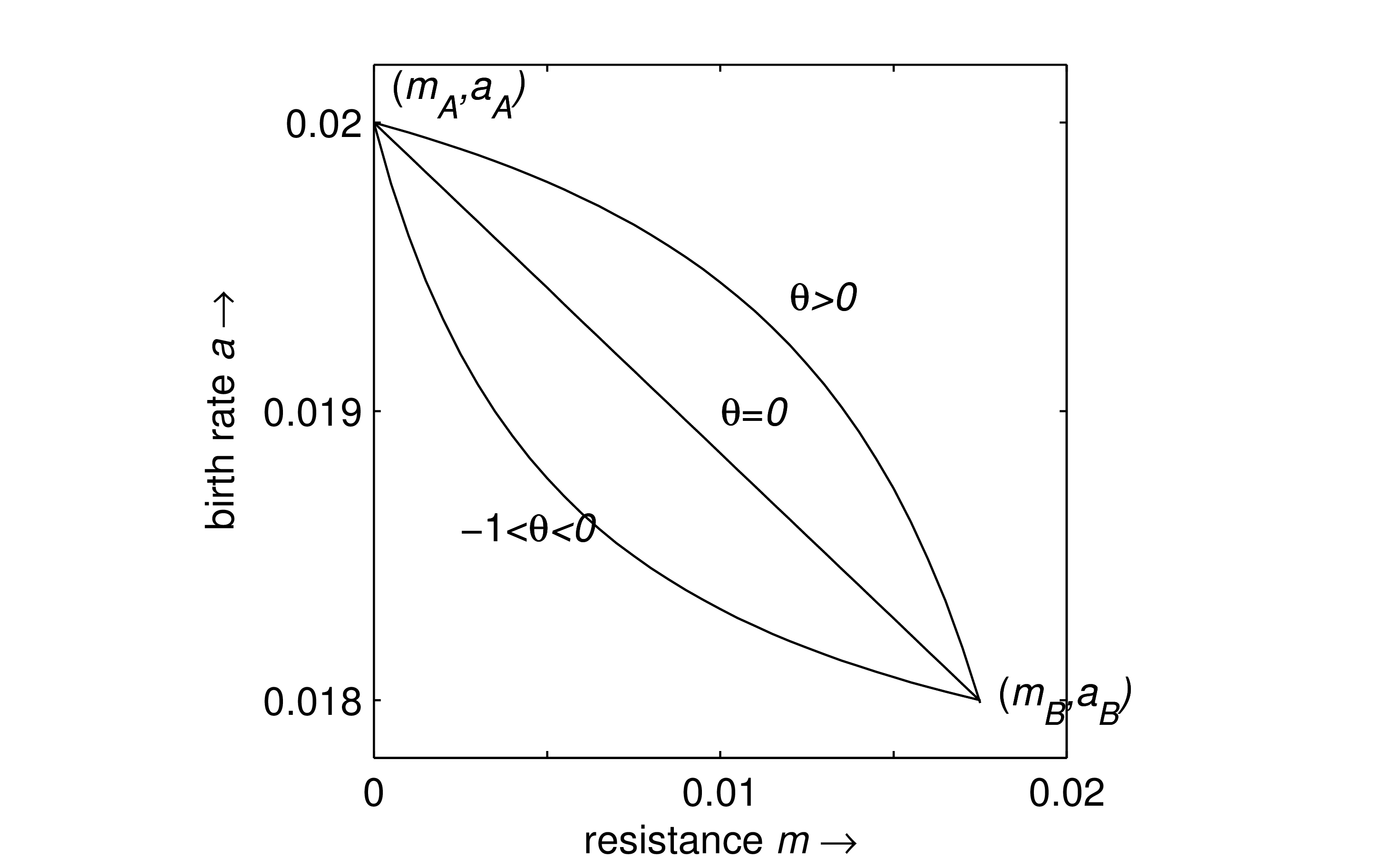

Supplement: S2 Fig — (TIF) [file pone.0169168.s002.tif]
