# Supplementary material for: Predicting the Potential for Natural Recovery of Atlantic Salmon (Salmo salar L.) Populations following the Introduction of Gyrodactylus salaris Malmberg, 1957 (Monogenea)
Source: PLoS One. 2016 Dec 29;11(12):e0169168. doi: 10.1371/journal.pone.0169168 (PMC5199095; doi:10.1371/journal.pone.0169168)
Supplement: S1 Appendix — (DOCX) [file pone.0169168.s003.docx]

**Appendix S1 - Derivation of the model**

Following the framework set out by Anderson & May (1) and May & Anderson (2) the basic model is constructed using the assumptions below. The model is also represented graphically in Fig S1.

**Fig S1**. Schematic representation of the Atlantic salmon-*Gyrodactylus salaris* interaction.

**Salmon growth**

The growth of the salmon population is determined by the reproductive rate, *a*, minus the natural mortality rate, *b*. Both rates are assumed to be constants. A density dependence constraint (A1), linearly proportional to host density, was added to the natural host mortality rate, *b*, to account for constraints such as predation, resource limitation, fishing, *etc*.

$$\hat{b}=b+sH (A1)$$

**Carrying Capacity**

Having density dependence in the model (A1) constrain the host population growth to a threshold, *K,* in the absence of parasitic infection (P = 0). This threshold is commonly referred to as the carrying capacity. The general form of the carrying capacity is defined as:

$$K= \frac{a-b}{s} (A1.2)$$

***Gyrodactylus salaris* induced salmon mortalities**

If the rate of *G. salaris* induced salmon deaths is assumed to be linearly proportional to the number of *G. salaris* parasites a salmon host harbours, with *α* a constant representing the pathogenicity of parasites to hosts, then the number of salmon mortalities in a small interval of time, *δt*, among salmon hosts with *i* parasites can be is given as *αiδt*. Thus, for a population of salmon of size *H(t)*, the total rate of loss of salmon hosts due to infection is:

$$\alpha H\left( t \right)=\sum_{t=0}^{\infty} ip\left( i \right) (A2)$$

Where $p(i)$ is the probability that a given host has *i* parasites. Now,

$$\sum_{t=0}^{\infty} ip(i)\equiv E_{t}\left( i \right)=\frac{P\left( t \right)}{H\left( t \right)}=mean parasite load (A3)$$

$$\Rightarrow\alpha H\left( t \right)=\sum_{t=0}^{\infty} ip(i)=\alpha H\left( t \right)\frac{P\left( t \right)}{H\left( t \right)} (A4)$$

$$=\alpha P\left( t \right) (A5)$$

***Gyrodactylus salaris* births**

The reproductive rate of parasites is defined as *µ*. This gives the net rate for the total *G. salaris* population as:

$$\mu H\left( t \right)=\sum_{t=0}^{\infty} ip(i)=\mu H\left( t \right)\frac{P\left( t \right)}{H\left( t \right)} (A6)$$

$$=\mu P\left( t \right) (A7)$$

***Gyrodactylus salaris* mortality**

Parasites can die due to natural host deaths, parasite induced host deaths and natural parasite deaths.

Via natural host deaths: Net rate of G. salaris mortality due to natural salmon mortality is

defined as:

$$bH\left( t \right)=\sum_{t=0}^{\infty} ip(i)=bH\left( t \right)\frac{P\left( t \right)}{H\left( t \right)} (A8)$$

$$=bP\left( t \right) (A9)$$

Via parasite induced host deaths: Net rate of *G. salaris* mortality due to *G. salaris* induced salmon mortality, assuming a Poisson distribution, is defined as:

$$\alpha H\left( t \right)=\sum_{t=0}^{\infty} i^{2}p\left( i \right)={\alpha H\left( t \right)E}_{t}\left( i^{2} \right) (A10)$$

$$=\alpha\frac{{(P\left( t \right))}^{2}}{H(t)}+\alpha P\left( t \right) (A11)$$

Via natural parasite deaths: Net rate of G. salaris mortality due to natural causes is defined as:

$$\varepsilon P\left( t \right) (A12)$$

**Detached parasites**

When a *G. salaris* parasite becomes detached from a salmon host it is able to survive for a period of time at low temperatures either drifting in the water column or settling on a substrate or dead host, before possibly infecting a new host (3–6). Parasite survival when off a host or on a dead host is, considerably lower than survival when on a host.

In the present study parasites become detached from a salmon host, either by a failed transfer to a new host or by being knocked/washed off, at rate *λ*, and thus leave the on-fish (attached) parasite population. Parasites can then re-enter the on-host parasite population by infecting salmon at rate *β*. Mortalities of parasites in the off-host population (*i.e.,* in the environment) occur at an increased rate, *σ*.

**Immune response**

Immune response to infection was added to the salmon-*G. salaris* system as follows:

- When an infection is present an immune response is mounted by the host. This happens at a rate, $m$ and is proportional to the number of parasites per host, *i.e.,* as the parasite infection level increases, the level of immunity also increases.
- The rate of change of parasites is negatively correlated with the rate of change of immunity in that the immune response causes an additional death rate in parasites, $\rho$.
- The immune response decays at a continuous rate, $\xi$.

Therefore, the host-parasite model describing the dynamics of salmon-*G. salaris* interactions with total on-host parasite numbers, assuming a Poisson distribution is as follows:

$$\frac{dH}{dt}=\left( a-b-sH \right)H-\alpha P \left( A13 \right)$$

$$\frac{dP}{dt}=P\left[ \left( \mu-\varepsilon-\rho I \right)-\lambda-b-sH-\alpha\left( 1+\frac{P}{H} \right) \right]+\beta WH \left( A14 \right)$$

$$\frac{dI}{dt}=m\frac{P}{H}-\xi I \left( A15 \right)$$

$$\frac{dW}{dt}=P\left[ \lambda+b+sH+\alpha\left( 1+\frac{P}{H} \right) \right]-\sigma W-\beta WH (A16)$$

Where, dH/dt is the rate of change of the salmon (host) population; dP/dt is the rate of change of the on-host *G. salaris* population; dI/dt is the rate of change of the immune response; dW/dt is the rate of change of the off-host *G. salaris* population.

We now re-evaluate the model in (A13-16) in terms of mean parasite burden per host (*i.e*., P/H) using the quotient rule:

$$\frac{d\frac{P}{H}}{dt}=\frac{H\frac{dP}{dt}-P\frac{dH}{dt}}{H^{2}} (A17)$$

$$=\frac{1}{H}\frac{dP}{dt}-\frac{P}{H^{2}}\frac{dH}{dt} (A18)$$

Now, equation (A14) implies,

$$\frac{1}{H}\frac{dP}{dt}=\frac{1}{H}\left[ P\left[ (\mu-\varepsilon-\rho I)-\lambda-b-sH-\alpha\left( 1+\frac{P}{H} \right) \right]+\beta WH \right] (A19)$$

$$=\frac{P}{H}\left[ \left( \mu-\varepsilon-\rho I-\lambda-b-sH-\alpha\right) \right]-\frac{\alpha P^{2}}{H^{2}}+\beta WH (A20)$$

Similarly, equation (A13) implies,

$$\frac{P}{H^{2}}\frac{dH}{dt}=\frac{P}{H^{2}}\left[ \left( a-b-sH \right)H-\alpha P \right] (A21)$$

$$=\frac{P}{H}\left( a-b-sH \right)-\alpha\frac{P^{2}}{H^{2}} (A22)$$

Therefore, substituting equations (A20) and (A22) into equation (A18) gives the following,

$$\frac{1}{H}\frac{dP}{dt}-\frac{P}{H^{2}}\frac{dH}{dt}=\frac{P}{H}\left( \mu-\varepsilon-\rho I-\lambda-\alpha-a \right)+\beta W (A23)$$

Thus, the model in terms of mean parasite burden, with $M=\frac{P}{H}$ is given by,

$$\frac{dH}{dt}=\left( a-b-sH \right)H-\alpha MH \left( A24 \right)$$

$$\frac{dM}{dt}=\left( \mu-\varepsilon-\rho I-\lambda-\alpha-a \right)M+\beta W \left( A25 \right)$$

$$\frac{dI}{dt}=mM-\xi I \left( A26 \right)$$

$$\frac{dW}{dt}=MH\left[ \lambda+b+sH+\alpha\left( 1+M \right) \right]-\sigma W-\beta WH (A27)$$
