# Supplementary material for: Predicting the Potential for Natural Recovery of Atlantic Salmon (Salmo salar L.) Populations following the Introduction of Gyrodactylus salaris Malmberg, 1957 (Monogenea)
Source: PLoS One. 2016 Dec 29;11(12):e0169168. doi: 10.1371/journal.pone.0169168 (PMC5199095; doi:10.1371/journal.pone.0169168)
Supplement: S2 Appendix — (DOCX) [file pone.0169168.s004.docx]

**Appendix S2 - Equilibrium and stability analysis of the model**

This appendix contains the algebraic stability analysis for the model in equation (2) in the main text. Where possible, models were analysed using the standard algebraic methods (1,2,7–10). In cases where solutions could not be found analytically, numerical solutions were obtained using appropriate mathematical computer software. Wolfram Mathematica (11) was the package used for simulating the various models in the present study. Solutions in Mathematica were obtained via the “NDSolve” function. The “NDSolve” function is used to find numerical solutions to ordinary differential equations.

**Equilibrium analysis**

To determine whether equilibria exist for a system of equations the standard methods of analysis are followed (1,2,7–10) with equilibria found by setting the equations in the model to zero and solving for H, M,W, I, *etc*.

Setting *dH/dt = dM/dt =dI/dt= dW/dt = 0* we find the following equilibria exist:

1. (H, M, I, W) = (0, 0, 0, 0) the trivial equilibrium with no salmon host or *G. salaris* parasites (neither on or off hosts), and hence, no immune response.
2. (H, M, I, W) = (K, 0, 0, 0) the disease-free equilibrium with salmon population growth in the absence of *G. salaris* infection. Again, no immune response is required/mounted in the absence of infection.
3. (H, M, I, W) = (H*, M*, I*, W*) the co-existence equilibrium == INTRACTABLE

**Stability of equilibria**

The standard methods of analysis, as outlined by Anderson & May (1); May & Anderson (2); Anderson & May (7), are employed to determine stability of equilibria. If small perturbations from equilibrium return to said equilibrium point (when certain conditions are met) then the system is locally stable. For each equilibrium value the resulting Jacobian matrix is calculated. From the Jacobian, the characteristic equation and eigenvalues are obtained. If the eigenvalues of the Jacobian have negative real parts then local stability of the equilibrium value is confirmed.

Jacobian matrices are of the form:

$$\boldsymbol{J}=\left( \begin{matrix} \frac{\partial H}{\partial H} & \frac{\partial H}{\partial M} & \frac{\partial H}{\partial I} & \frac{\partial H}{\partial W} \\ \frac{\partial M}{\partial H} & \frac{\partial M}{\partial M} & \frac{\partial M}{\partial I} & \frac{\partial M}{\partial W} \\ \frac{\partial I}{\partial H} & \frac{\partial I}{\partial M} & \frac{\partial I}{\partial I} & \frac{\partial I}{\partial W} \\ \frac{\partial W}{\partial H} & \frac{\partial W}{\partial M} & \frac{\partial W}{\partial I} & \frac{\partial W}{\partial W} \end{matrix} \right)$$

Thus, the general form of the Jacobian matrix for the model in (2) is as follows:

$$\boldsymbol{J}=\left( \begin{matrix} a-b-\alpha M^{*}-2sH^{*} & -\alpha H^{*} & 0 & 0 \\ 0 & \mu-(a+\alpha+\epsilon+\rho I^{*}+\lambda) & \rho M^{*} & \beta\\ 0 & m & -\xi& 0 \\ M^{*}(\alpha M^{*} +\alpha+b+\lambda+2sH^{*})-\beta W^{*} & H^{*}(2\alpha M^{*}+\alpha+b+sH+\lambda) & 0 & -\sigma-\beta H^{*} \end{matrix} \right)$$

1. Eigenvalues of Jacobian at (H*, M*, I*, W*) = (0, 0, 0, 0) are given by,

$$\left| \begin{matrix} a-b-\Lambda& 0 & 0 & 0 \\ 0 & \mu-(a+\alpha+\epsilon+\lambda)-\Lambda& 0 & \beta\\ 0 & m & -\xi-\Lambda& 0 \\ 0 & 0 & 0 & -\sigma-\Lambda\end{matrix} \right|=0$$

With eigenvalues: $a-b$, $\mu-(a+\alpha+\epsilon+\lambda)$, $-\xi, -\sigma$. This equilibrium is only stable if

$$a<b \to Host births<Host deaths$$

$$\mu<(a+\alpha+\epsilon+\lambda) \to Parasite births<Parasite deaths$$

1. Eigenvalues of Jacobian at (H*, M*, I*, W*) = (K, 0, 0, 0), with K=(a-b)/s, are given by,

$$\left| \begin{matrix} -a+b-\Lambda& -\alpha K & 0 & 0 \\ 0 & \mu-(a+\alpha+\epsilon+\lambda)-\Lambda& 0 & \beta\\ 0 & m & -\xi-\Lambda& 0 \\ 0 & K(\alpha+a+\lambda) & 0 & -\sigma-\beta K-\Lambda\end{matrix} \right|=0$$

With two eigenvalues being: $-a+b$ and $-\xi$. The other two are found from:

$$\left| \begin{matrix} \mu-(a+\alpha+\epsilon+\lambda)-\Lambda& \beta\\ K(\alpha+a+\lambda) & -\sigma-\beta K-\Lambda\end{matrix} \right|=0$$

$$\Lambda^{2}-\left[ \underset{<0}{\underbrace{\mu-\left( a+\alpha+\epsilon+\lambda\right)-\sigma-\beta K}} \right]\Lambda-\left[ \underset{<0}{\underbrace{\left( \mu-\epsilon\right)\beta K+\sigma\left( \mu-\left( a+\alpha+\epsilon+\lambda\right) \right)}} \right] =0$$
