# Supplementary material for: Predicting the Potential for Natural Recovery of Atlantic Salmon (Salmo salar L.) Populations following the Introduction of Gyrodactylus salaris Malmberg, 1957 (Monogenea)
Source: PLoS One. 2016 Dec 29;11(12):e0169168. doi: 10.1371/journal.pone.0169168 (PMC5199095; doi:10.1371/journal.pone.0169168)
Supplement: S3 Appendix — (DOCX) [file pone.0169168.s005.docx]

**Appendix S3 - Derivation of the fitness of mutant type & Trade-off**

Consider a resident host strain, with immune response, with population $H$ existing alone in an environment, with the dynamics as given in equation (2) in the main paper. Now suppose a mutation creates a host with slightly different immune trait, given by $\hat{m}$, with population $\hat{H}$. The dynamics of this new type are given by

$$\frac{d\hat{H}}{dt}=\left( a(\hat{m})-b-s(H+\hat{H}) \right)\hat{H}-\alpha\hat{M}\hat{H}$$

$$\frac{d\hat{M}}{dt}=\left( \mu-\varepsilon-\hat{I}-\lambda-\alpha-a(\hat{m}) \right)\hat{M}+\beta W$$

$$\frac{d\hat{I}}{dt}=\hat{m}\hat{M}-\xi\hat{I}$$

where the $\hat{M}$ and $\hat{I}$ are the parasite burden and immune response for this mutant host strain.

As the parasite generation time is much shorter than the host, we make the assumption that the parasites reach their average burden much. This allows is to simplify the analytical work by allowing us to take $\hat{M}$ to be the parasite burden equilibrium density on a mutant host $\hat{M}_{eq}$, found by solving the equations for $d\hat{M}/dt=d\hat{I}/dt=0$, and is given by

$$\hat{M}_{eq}\left( \hat{m},W\left( m \right) \right)=\frac{\xi\left( \delta-a\left( \hat{m} \right) \right)+\sqrt{\xi^{2}\left( \delta-a\left( \hat{m} \right) \right)^{2}+4\hat{m}\beta\xi W(m)}}{2\hat{m}}$$

where $\delta=\mu-\varepsilon-\lambda-\alpha$. Now the fitness of this mutant host, $r\left( \hat{m},m \right),$ can be derived from the mutant host equation

$$\frac{d\hat{H}}{dt}=\left[ a(\hat{m})-b-s(H+\hat{H})-\alpha\hat{M}_{eq} \right]\hat{H}$$

where, if we assume the mutant is initially rare, i.e. $\hat{H}\approx0$, the fitness is

$$r\left( \hat{m},m \right)=a(\hat{m})-b-sH(m)-\alpha\hat{M}_{eq}(\hat{m},W(m))$$

If we start from a fully susceptible host strain (Atlantic salmon), then we do not expect a single mutation to create a highly resistant (Baltic) strain. Instead it would take a series of smaller mutation and replacement events to eventually get to that high level of $m$. We can predict which direction and how far the immune response, $m$, will actually evolve using the fitness gradient

$$\left. \frac{\partial r}{\partial\hat{m}} \right|_{\hat{m}=m}=\left. a^{'}\left( \hat{m} \right) \right|_{\hat{m}=m}-\alpha\left. \frac{\partial\hat{M}}{\partial\hat{m}} \right|_{\hat{m}=m}$$

If this fitness gradient is positive, them $m$ will increase, whereas if it is negative $m$ will decrease. Evolution eventually stops when $m$ reaches an evolutionary singular point, as defined where the fitness gradient is zero. If this point is stable, it will stop evolving here and any mutations will always have negative fitness. The exact values of these singular points can be solved by solving the fitness gradient being zero for $m$.

**The trade-off**

Given we have two ‘known’ host types: the Atlantic salmon, with immune response $m_{A}$ and birth rate $a_{A}$, and Baltic salmon, with immune response $m_{B}$ and birth rate $a_{B}$, we choose a function which passes through those two points and has a varying curvature between them, as defined by $\theta$. The particular function we choose is

$$a\left( m \right)=a_{A}-\frac{(a_{A}-a_{B})\left( 1-\frac{m-m_{B}}{m_{A}-m_{B}} \right)}{1+\theta\frac{m-m_{B}}{m_{A}-m_{B}}}$$

Here $\theta>0$ represents an ‘accelertaingly costly trade-off’, whereby each benefit (*i.e.,* an increase in immune response, $m$) comes at an increasing (accelerating) cost (*i.e.,* a larger decrease in birth rate, $a$). Conversely, $\theta<0$ represents a ‘decelertaingly costly trade-off’, whereby each benefit comes at an decreasing (decelerating) cost (*i.e.,* a smaller decrease in birth rate, $a$). Finally $\theta=0$ represents a linear trade-off, whereby each benefit always comes at the same cost (12). Plots of these are shown in Fig S2.

**Fig S2:** The trade-off between host birth rate, $a$, and the rate hosts mount an immune response to the parasite (resistance), $m$. The functional form is given in equation (3). An ‘acceleratingly costly trade-off’ is defined when benefits (increases in immune response, $m$) are met with accelerating costs (larger decreases in birth rate, $a$). Conversely we define ‘deceleratingly costly trade-offs’ when the costs decelerate.
