# Supplementary material for: Predicting the Potential for Natural Recovery of Atlantic Salmon (Salmo salar L.) Populations following the Introduction of Gyrodactylus salaris Malmberg, 1957 (Monogenea)
Source: PLoS One. 2016 Dec 29;11(12):e0169168. doi: 10.1371/journal.pone.0169168 (PMC5199095; doi:10.1371/journal.pone.0169168)
Supplement: S4 Appendix — (DOCX) [file pone.0169168.s006.docx]

**Appendix S4 - Details of evolutionary simulations**

1) Start with a monomorphic population, *i.e.* a single strain of the host, with an immune response value $m$ (initially taken as 0.0018 in all simulations.) Run the model, given in equation (2) in main paper, for a time tf.

2) If any of the final densities are below a (low) threshold then set them to zero. For each positive host population, record the final density values and define them as *H_res_*. (If there are more than 1 host strain, label them *H_res1_*, *H_res2_* *etc*).

3) Label the existing strain as $m_{res}$. (If there are more than 1 strain, label them $m_{res1}$, $m_{res2}$ *etc*)

4) Create a mutant strain by drawing a number at random from a normal distribution with mean $m_{res}$ and standard deviation $\sigma_{mut}$ (taken as 0.0001 in all simulations). Label this value as $m_{mut}$. If there is more than one host strain present, randomly select from which strain the mutation will occur (with appropriate bias for population size and birth rates – *i.e.* the larger the population and/or more births, the more likely it is the mutation will come from that population).

5) Add equations for this mutant host type to the existing equations, creating an (*n*)-strain model (where $n$ is the number of trait values, *i.e.* 1 mutant and *n-1* resident values). For the initial densities, we take $m_{res}$ to have densities $H_{res}$, and $m_{mut}$ to have “low” density.

6) Run the multi-strain model below, with the $n$ strains present, for a time tf.

$$\frac{dH_{i}}{dt}=\left( a\left( m_{i} \right)-b-s\sum_{j=1}^{n} H_{j} \right)H_{i}-\alpha M_{i}H_{i}$$

$$\frac{dM_{i}}{dt}=\left( \mu-\varepsilon-\rho I_{i}-\lambda-\alpha-a(m_{i}) \right)M_{i}+\beta W$$

$$\frac{dI_{i}}{dt}=m_{i}M_{i}-\xi I_{i}$$

$$\frac{dW}{dt}=\sum_{i=1}^{n} M_{i}H_{i}\left[ \lambda+b+s\sum_{j=1}^{n} H_{j}+\alpha\left( 1+M_{i} \right) \right]-\sigma W-\sum_{i=1}^{n} \beta WH_{i}$$

7) Repeat from stage 2, until evolutionary and population behaviour have both settled.
