# Supplementary material for: Predicting the Potential for Natural Recovery of Atlantic Salmon (Salmo salar L.) Populations following the Introduction of Gyrodactylus salaris Malmberg, 1957 (Monogenea)
Source: PLoS One. 2016 Dec 29;11(12):e0169168. doi: 10.1371/journal.pone.0169168 (PMC5199095; doi:10.1371/journal.pone.0169168)
Supplement: S5 Appendix — (DOCX) [file pone.0169168.s007.docx]

**Appendix S5 - Supplementary Information reference list**

1. Anderson RM, May RM. Regulation and stability of host-parasite population interactions. I: Regulatory processes. J Anim Ecol. 1978; 47:219–47.

2. May RM, Anderson RM. Regulation and stability of host-parasite population interactions. II: Destabilizing processes. J Anim Ecol. 1978; 47:249–67.

3. Bakke TA, Harris PD, Jansen PA, Hansen LP. Host specificity and dispersal strategy in gyrodactylid monogeneans, with particular reference to *Gyrodactylus salaris* (Platyhelminthes, Monogenea. Dis Aquat Organ. 1992; 13:63–74.

4. Bakke TA, Cable J, Harris PD. The biology of gyrodactylid monogeneans: The “Russian-doll killers.” Adv Parasitol. 2007; 64:161–460.

5. Olstad K, Cable J, Robertsen G, Bakke TA. Unpredicted transmission strategy of *Gyrodactylus salaris* (Monogenea : Gyrodactylidae): survival and infectivity of parasites on dead hosts. Parasitology. 2006; 133:33–41.

6. Soleng A, Jansen PA, Bakke TA. Transmission of the monogenean *Gyrodactylus salaris*. Folia Parasitol (Praha). 1999; 46:179–84.

7. Anderson RM, May RM. The population dynamics of microparasites and their invertebrate hosts. Philos Trans R Soc Lond B Biol Sci. 1981; 291:451–524.

8. Anderson RM, May RM. Infectious diseases of humans: Dynamics and control. Oxford: Oxford University Press; 1991.

9. Murray JD. Mathematical Biology : I . An Introduction (3rd Ed). Springer; 2002. 576 p.

10. Murray JD. Mathematical Biology II: Spatial Models and Biomedical Applications (3rd Ed). Springer; 2003. 811 p.

11. Mathematica Research Inc. Mathematica, Version 7.0, Wolfram Research, Inc., Champaign, Illinois. 2008.

12. Hoyle A, Bowers RG, White A, Boots M. The influence of trade-off shape on evolutionary behaviour in classical ecological scenarios. J Theor Biol. 2008; 250:498–511.
